# Effectiveness of romosozumab following prior raloxifene treatment in primary osteoporosis: An observational study

**DOI:** 10.1016/j.bonr.2026.101900

**Published:** 2026-01-26

**Authors:** Kazuaki Mineta, Toshihiko Nishisho, Masahiko Okada, Mitsuhiro Kamada, Koichi Sairyo

**Affiliations:** a Department of Orthopaedic Surgery, Tokushima Kensei Hospital, 4-9 Shimosuketo-cho, Tokushima, 770-8547, Japan; b Department of Orthopedics, Institute of Biomedical Sciences, Tokushima University Graduate School, 3-18-15 Kuramoto-cho, Tokushima, 770-8503, Japan

**Keywords:** Romosozumab, Raloxifene, Bone mineral density, Osteoporosis, Bone turnover markers

## Abstract

Romosozumab is an anti-sclerostin antibody that increases bone formation and decreases bone resorption. It has been available for patients at high risk of osteoporotic fractures in Japan since 2019. The aim of this study was to clarify the clinical effectiveness and safety of romosozumab following previous treatment with raloxifene. The study had an observational pre–post design and included 62 women with primary osteoporosis. Romosozumab 210 mg was administered subcutaneously every 4 weeks for 12 months in patients who had been previously treated with raloxifene (raloxifene group, *n* = 12) and in those who were treatment-naïve (treatment-naïve group, *n* = 50). The incidence of new fractures, safety, and changes in bone mineral density (BMD) and bone turnover markers (BTMs) were recorded. No new fractures occurred in either group. Ten patients (16.1%) in the treatment-naïve group discontinued romosozumab for the following reasons: non-serious adverse events (*n* = 2, 3.2%), a change to another hospital (*n* = 1, 1.6%), self-discontinuation (*n* = 5, 8.1%), and financial constraints (*n* = 2, 3.2%). The percent changes in spine BMD and total hip BMD at 12 months were respectively +13.5% and + 4.9% in the treatment-naïve group and + 16.0% and + 3.4%, respectively, in the raloxifene group. We did not detect significant differences in the changes in BTMs according to whether there was previous treatment with raloxifene. Prior raloxifene treatment may be well tolerated and may not affect increases in BMD, changes in BTMs, and fracture prevention in romosozumab therapy.

## Highlights

• BMD gains may not be attenuated in romosozumab therapy following prior raloxifene.
• BTMs may not change according to whether there was prior raloxifene treatment.
• Romosozumab following raloxifene may be safe in primary osteoporosis.
• The new fracture rate may be very low in romosozumab therapy after raloxifene.

## 1 Introduction

The number of patients with osteoporosis is increasing in developed countries worldwide as a result of population aging (Yoshimura et al., 2010). Approximately 35% of all elderly women have osteoporosis (Salari et al., 2021), and 178 million new osteoporosis-related fractures were reported worldwide in 2019 (GBD 2019 Fracture collaborators, 2021). Osteoporosis imposes a significant economic and societal burden, and fragility fractures are painful and lower quality of life (Lewiecki et al., 2019; Lips and van Schoor, 2005).

Drug therapy is the cornerstone of osteoporosis treatment and many drugs are available. Romosozumab is an anti-sclerostin antibody that first became available in clinical practice in Japan in March 2019 for patients with osteoporosis at high risk of fracture (Soen et al., 2013). Romosozumab inhibits the suppression of Wnt signaling and has the dual effect of promoting bone formation and decreasing bone resorption (Ominsky et al., 2015). The effectiveness and safety of this agent have been demonstrated in pivotal studies (Cosman et al., 2016; Ishibashi et al., 2017). However, several recent real-world studies (Inose et al., 2022; Kobayakawa et al., 2021; Tominaga et al., 2021; Ebina et al., 2020) have suggested that the effectiveness of romosozumab in patients with a history of treatment with antiresorptive drugs may be inferior to that in those who have not previously received these medications. Raloxifene has antiresorptive activity (Delmas et al., 2002) and has demonstrated safety in long-term use (Messalli and Scaffa, 2010). However, there is no published research on the effectiveness of romosozumab following prior treatment with raloxifene.

The aim of this study was to elucidate the effectiveness of romosozumab for primary osteoporosis in patients previously treated with raloxifene by investigating the actual clinical effects, adverse events, and the percent change in bone mineral density (BMD) from baseline and the kinetics of bone turnover markers (BTMs) during 12 months of treatment.

## 2 Materials and methods

### 2.1 Study design and participants

The study had a prospective pre–post observational design. and included patients who were started on romosozumab for primary osteoporosis at Tokushima Kensei Hospital between March 2019 and June 2022.

All patients received a subcutaneous injection of romosozumab 210 mg on entry into the study and monthly thereafter. Patients were eligible for inclusion in the study if they had a high risk of fracture, defined as BMD ≤ −2.5 standard deviations (SDs) with at least one fragility fracture, lumbar spine BMD < −3.3 SDs, at least two previous vertebral fractures, or a semiquantitative assessment score for post-vertebral fracture of grade 3, as defined by the Japan Osteoporosis Society (Japan Osteoporosis Society, 2015). All osteoporosis patients with a high risk of fracture were started on romosozumab soon after it became available for clinical use in Japan in March 2019. Patients who were previously treated with raloxifene were switched to romosozumab when their prescription was renewed (the raloxifene group), and those who were treatment-naïve at the time of diagnosis were started on romosozumab (the treatment-naïve group). Patients who had experienced a cardiovascular event within the preceding year were excluded, as were those with known underlying diseases that could lead to secondary osteoporosis, those with a history of steroid use, and those with cancer. All patients received a vitamin D3 supplement (25.0 μg/day) throughout the study period and were encouraged to have an adequate intake of calcium from a well-balanced diet.

The study was approved by the Ethics Review Committee at Kensei Hospital (approval number 2503) and conducted in accordance with the principles of the Declaration of Helsinki. Verbal informed consent was obtained from all patients, and this was documented in the medical records.

### 2.2 Study outcomes

The study was designed as a pre–post comparison of study endpoints. The primary endpoints were the discontinuation rate, reasons for discontinuation, and adverse events. Information on adverse events was obtained from the interview at the point of discontinuation.

Secondary endpoints included patient characteristics, changes in BMD and serum BTMs, and the incidence of new fractures during 12 months of treatment with romosozumab. Changes in BMD were assessed by dual-energy X-ray absorptiometry using a PRODIGY Fuga-C densitometer (GE Healthcare, Tokyo, Japan). Areal BMD at the lumbar spine (L1–L4) and total hip was assessed at baseline and after 6 and 12 months of treatment with romosozumab. Previous fracture sites and sites at which orthopedic surgery had been performed were not evaluated. Data for patients who developed a bone fracture or underwent orthopedic surgery during the study were excluded from the BMD analysis. Serum BTMs were measured in the morning before administration of romosozumab at the start of treatment and 1, 3, 6, and 12 months later. Procollagen type 1 N-propeptide (P1NP) was used to assess bone formation and tartrate-resistant acid phosphatase 5b (TRACP-5b) for indirect assessment of bone resorption (Gerz et al., 1998; Igarashi et al., 2002). The minimum significant change (MSC) in the percent value from baseline was 12.1% for P1NP and 12.4% for TRACP-5b (Nishizawa et al., 2019). Clinical fractures were identified through monitoring of clinical symptoms and confirmed radiographically by the physicians.

### 2.3 Statistical analysis

Patient background characteristics are expressed as the mean ± standard deviation. P1NP and TRACP-5b are shown as the median [interquartile range]. Percent changes and changes from baseline to the study time points for BMD, P1NP, and TRACP-5b were assessed using repeated-measures ANOVA to test for significant associations with time and a time × group (treatment-naïve vs. pretreatment with raloxifene) interaction. When a significant time effect was observed, the Bonferroni test for post hoc comparisons was used to identify significant differences between the study time points. The Mann–Whitney *U* test was used to evaluate differences between the groups win percent changes and changes from baseline to the study time points for BMD and BTMs. Data for 4 patients in the treatment-naïve group who underwent orthopedic surgery at bilateral hip before the study were excluded from the total hip BMD analysis. All statistical analyses were performed using EZR (Kanda, 2013) (Saitama Medical Center, Jichi Medical University, Saitama, Japan), which is a graphical user interface for R (R Foundation for Statistical Computing, Vienna, Austria). More precisely, it is a modified version of R commander designed to add statistical functions frequently used in biostatistics. *P*-values of <0.05 were considered statistically significant.

## 3 Results

### 3.1 Characteristics of patients, discontinuations, and adverse events

A total of 62 female patients were initially enrolled. During the study period, 10 patients (16.1%) in the treatment-naïve group discontinued romosozumab for the following reasons: non-serious adverse events (*n* = 2, 3.2%), a change to another hospital (*n* = 1, 1.6%), self-discontinuation (*n* = 5, 8.1%), and financial constraints (n = 2, 3.2%). The adverse events included an injection site reaction (*n* = 1, 1.6%) and fever (n = 1, 1.6%), both of which occurred at the first administration of romosozumab. No patients in the raloxifene group discontinued romosozumab. There were no cardiovascular events or cases of severe hypocalcemia, atypical fracture, or osteonecrosis of the jaw during treatment. Data of patients who discontinued romosozumab were excluded from the evaluation of effectiveness.

The remaining 52 patients completed 12 months of treatment with romosozumab and were included in all subsequent analyses. The remaining 52 patients reported no adverse events. Patient demographics and clinical characteristics at baseline are shown in Table 1. Twelve patients (23.1%) had a history of treatment for osteoporosis with raloxifene. No patients had been treated with a bisphosphonate or other anti-osteoporosis drug before raloxifene, and none had received estrogen therapy. Seven patients (13.5%) who were treated with raloxifene used vitamin D concomitantly before initiation of romosozumab. All patients took a vitamin D supplement during treatment with romosozumab.

**Table 1:** Patient demographics and clinical characteristics at baseline.

Variable	*N* = 52
Age (years)	76.0 ± 7.7
Body mass index⁎	21.5 ± 3.1
Bone mineral density (g/cm^2^) (T score)	
Lumbar spine	0.80 ± 0.14 (−2.5 ± 1.0)
Total hip	0.64 ± 0.10 (−2.4 ± 0.8)
Previous vertebral fracture	41 (78.8)
Previous hip fracture	4 (7.7)
Previous treatment with raloxifene for osteoporosis	12 (23.1)
Serum total P1NP (μg/L)	65.1 [17.6, 196]
Serum TRACP-5b (mU/dL)	421.7 [169, 800]
Serum albumin (g/dL)	4.1 ± 0.3
Serum corrected calcium (mg/dL)	9.3 ± 0.4
eGFR (mL/min/1.73 m^2^)	67.8 ± 9.0
Serum 25-hydroxyvitamin D (ng/mL)	15.5 ± 7.6

Data are shown as the mean ± standard deviation, median [interquartile range], or number (percentage) as appropriate. *eGFR* estimated glomerular filtration rate, *P1NP* procollagen type 1 N-propeptide, *TRACP-5b* tartrate-resistant acid phosphatase 5b.

⁎ Calculated as weight in kilograms divided by the square of height in meters.

### 3.2 Baseline characteristics of patients

Table 2 shows the baseline characteristics in the treatment-naïve group (*n* = 40) and the raloxifene group (*n* = 12). The average duration of raloxifene use was 24.3 ± 13.8 months. There were no statistically significant differences in any of the demographic or clinical variables, apart from the mean serum 25-hydroxyvitamin D level, which was significantly higher in the raloxifene group (*P* < 0.001).

**Table 2:** Clinical characteristics in the treatment-naïve group and the raloxifene group.

Variable	Treatment-naïve group	Raloxifene group
Number	40	12
Age (years)	75.1 ± 8.3	79.1 ± 4.7
Body mass index^a^	21.3 ± 2.6	22.1 ± 4.6
BMD (g/cm^2^) (T score)		
Lumbar spine	0.82 ± 0.13 (−2.4 ± 0.9)	0.75 ± 0.15 (−2.9 ± 1.1)
Total hip	0.65 ± 0.09 (−2.4 ± 0.7)	0.64 ± 0.12 (−2.5 ± 0.9)
Total P1NP (μg/L)	65.0 [17.6, 196]	65.6 [33.4, 145]
TRACP-5b (mU/dL)	446.9 [174, 800]	366.9 [169, 752]
Albumin (g/dL)	4.1 ± 0.3	4.1 ± 0.3
Calcium (mg/dL)	9.3 ± 0.4	9.3 ± 0.3
eGFR (mL/min/1.73 m^2^)	68.7 ± 9.4	65.1 ± 7.3
Serum 25-hydroxyvitamin D (ng/mL)	13.1 ± 5.5	23.5 ± 8.0⁎

Data are shown as the mean ± standard deviation, median [interquartile range], or number (percentage) as appropriate. ^a^Calculated as weight in kilograms divided by the square of height in meters. *BMD* bone mineral density, *eGFR* estimated glomerular filtration rate, *P1NP* procollagen type 1 N-propeptide, *TRACP-5b* tartrate-resistant acid phosphatase 5b.

⁎ *P* < 0.05, Mann–Whitney *U* test.

### 3.3 Effects of romosozumab on spine BMD and total hip BMD

Spine BMD significantly increased in a time-dependent manner, by 9.6% ± 6.0% after 6 months of treatment (*P* < 0.001) and by 14.1% ± 7.5% after 12 months of treatment (*P* < 0.001). The time × group interaction was not significantly associated with these changes in spine BMD (*P* = 0.160). Total hip BMD significantly increased in a time-dependent manner, by 3.3% ± 3.8% after 6 months (*P* < 0.001) and by 4.5% ± 5.3% after 12 months (P < 0.001). The time × group interaction was not significantly associated with these changes in total hip BMD (*P* = 0.860).

**Fig. 1:**
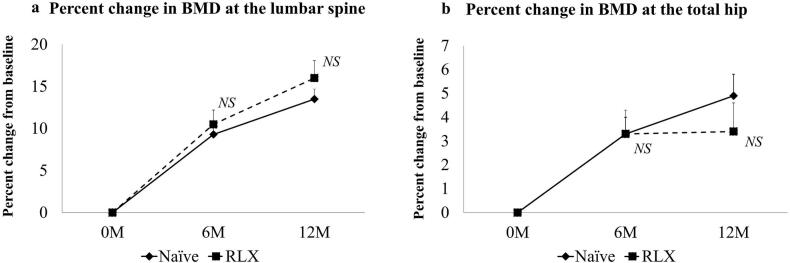
Comparison of percent changes in BMD in the treatment-naïve group and the raloxifene group. (a) Changes in BMD at the lumbar spine. (b) Changes in total hip BMD. There were no significant differences in the changes in BMD at the lumbar spine or at the total hip between the groups. *BMD* bone mineral density, *Naïve* treatment-naïve group, *RLX* raloxifene group.

Percent changes in BMD were compared between the treatment-naïve and raloxifene groups, and the data are summarized at Table 3. Spine BMD significantly increased in a time-dependent manner, by 9.3% ± 6.1% (*P* < 0.001) at 6 months and by 13.5% ± 7.5% (P < 0.001) at 12 months in the treatment-naïve group and by 10.5% ± 5.8% (*P* < 0.001) and 16.0% ± 7.4% (P < 0.001), respectively, in the raloxifene group. There were no significant differences between the groups at 6 months (*P* = 0.434) or 12 months (*P* = 0.396) (Fig. 1a).

**Table 3:** BMD changes in the treatment-naïve group and the raloxifene group.

Groups	Spine BMD (g/cm^2^)			p	TH BMD (g/cm^2^)			p
	0 M	6 M	12 M		0 M	6 M	12 M	
Naïve	0.82 ± 0.13	0.89 ± 0.12⁎1	0.92 ± 0.12⁎2,^3^	< 0.001	0.65 ± 0.09	0.66 ± 0.09†1	0.68 ± 0.09†2,^3^	< 0.001
RLX	0.75 ± 0.15	0.83 ± 0.18⁎1	0.86 ± 0.18⁎2	< 0.001	0.64 ± 0.12	0.66 ± 0.12†^4^	0.66 ± 0.12†^5^	0.003
P	0.228	0.434	0.396		0.721	0.805	0.502	

Data are shown as the mean ± standard deviation.

⁎^1^ : *p* < 0.05 Spine BMD (0 M) vs. Spine BMD (6 M).

⁎^2^ : *p* < 0.05 Spine BMD (0 M) vs. Spine BMD (12 M).

⁎^3^ : p < 0.05 Spine BMD (6 M) vs. Spine BMD (12 M).

†^1^ : p < 0.05 TH BMD (0 M) vs. TH BMD (6 M).

†^2^ : p < 0.05 TH BMD (0 M) vs. TH BMD (12 M).

†^3^ : p < 0.05 TH BMD (6 M) vs. TH BMD (12 M); Bonferroni post hoc test after repeated-measures ANOVA, *P* < 0.05; Mann-Whitney *U* test. *BMD* bone mineral density, *Naïve* treatment-naïve group, *M* month(s), *TH* total hip, *RLX* raloxifene group.

Total hip BMD significantly increased in a time-dependent manner, by +3.3% ± 4.0% at 6 months (*P* < 0.001) and by +4.9% ± 5.6% at 12 months (P < 0.001) in the treatment-naïve group and by +3.3% ± 3.4% (*P* = 0.018) and + 3.4% ± 4.2% (*P* = 0.031), respectively, in the raloxifene group. There were no significant differences between the groups at 6 months (*P* = 0.805) or 12 months (*P* = 0.502) (Fig. 1b).

### 3.4 Changes in BTMs during 12 months of treatment with romosozumab

**Fig. 2:**
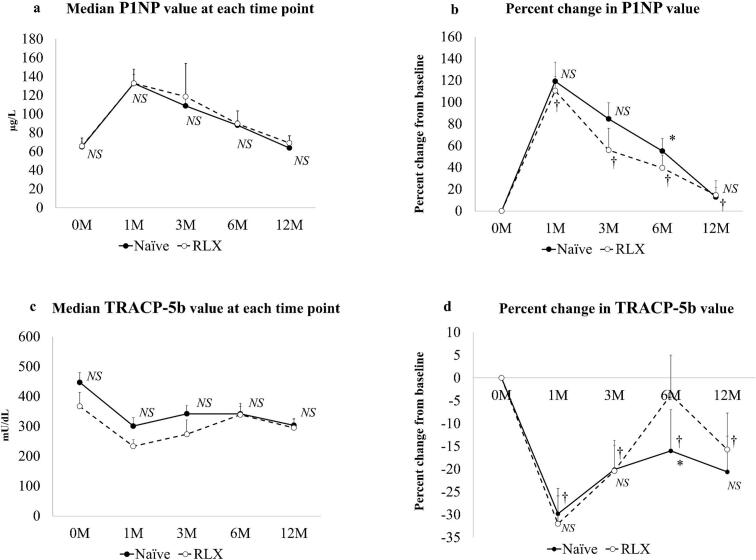
Serum bone turnover markers after treatment with romosozumab in the treatment-naïve group and the raloxifene group. (a) Median serum P1NP levels. (b) Percent changes in P1NP from baseline. (c) Median serum TRACP-5b levels. (d) Percent changes in TRACP-5b levels from baseline. *P < 0.05, Mann–Whitney *U* test. ^†^Percent change in the P1NP or TRACP-5b level from baseline exceeds the LSC. *LSC* least significant change, *Naïve* treatment-naïve group, *P1NP* procollagen type 1 N-propeptide, *RLX* raloxifene group, *TRACP-5b* tartrate-resistant acid phosphatase 5b.

The serum P1NP level and percent change from baseline were evaluated at each assessment time point according to pretreatment status (Table 4, Fig. 2a, b). The serum P1NP level significantly increased in a time-dependent manner from baseline to 6 months (*P* < 0.001). The time × group interaction was not significantly associated with changes in P1NP (*P* = 0.973). There was no significant difference in the serum P1NP level in either group at any time point (Table 4, Fig. 2a). The percent change from baseline showed the largest increase above the MSC (12.1%) (Nishizawa et al., 2019) at 1 month in both groups, and gradually decreased thereafter. The percent change in the P1NP level from baseline was significantly greater in the treatment-naïve group than in the raloxifene group at 6 months (*P* = 0.044); there was no statistically significant difference in the percent change in the P1NP level between the two groups at other time points (Table 4, Fig. 2b).

**Table 4:** P1NP changes in the treatment-naïve group and the raloxifene group.

Groups	P1NP value (μg/L)					p
	Percent change in P1NP value from baseline (%)					
	0 M	1 M	3 M	6 M	12 M	
Naïve	65.0 [17.6, 196]	132.7 [31.9, 291]⁎1	108.7 [46.1, 294]⁎2	88.0 [44.4, 185] ⁎3⁎4	63.8 [36.3, 133]	< 0.001
		119.2 ± 110.0	84.6 ± 91.8	55.1 ± 73.9	12.8 ± 55.5	
RLX	65.6 [33.4, 145]	132.8 [63.7, 268]⁎1	118.6 [42.9, 271]⁎2	89.6 [43.9, 215] ⁎3	69.0 [37.3, 107]	< 0.001
		110.6 ± 45.1	55.9 ± 49.4	83.1 ± 55.6⁎	14.6 ± 45.8	
P	0.957	0.938	0.694	0.625	0.633	
		0.767	0.536	0.044	0.541	

Data are shown as the mean ± standard deviation and the median [interquartile range].

⁎^1^ : p < 0.05 0 M vs. 1 M.

⁎^2^ : p < 0.05 0 M vs. 3 M.

⁎^3^ : p < 0.05 0 M vs. 6 M.

⁎^4^ : p < 0.05 0 M vs. 12 M; Bonferroni post hoc test after repeated-measures ANOVA.

⁎ P < 0.05; Mann-Whitney U test. *Naïve* treatment-naïve group, *M* month(s), *P1NP* procollagen type 1 N-propeptide, *RLX* raloxifene group.

The serum TRACP-5b level and percent changes from baseline were evaluated at each time point compared with before treatment (Table 5, Fig. 2c, d). The serum TRACP-5b level significantly decreased in a time-dependent manner from baseline to 6 months in the treatment-naïve group (*P* < 0.001) and significantly decreased at only 1 month in the raloxifene group (*P* = 0.005). The time × group interaction was not significantly associated with changes in TRACP-5b (*P* = 0.973). There was no significant difference between the groups in the serum TRACP-5b level at any time point (Table 5, Fig. 2c). The percent change from baseline showed the largest decrease above the MSC (12.4%) (Nishizawa et al., 2019) at 1 month in both groups, but gradually increased while maintaining a decrease above the MSC from 1 month onwards except at 6 months in the raloxifene group. The percent change in the TRACP-5b level from baseline was significantly greater in the treatment-naïve group than in the raloxifene group at 6 months (*P* = 0.045); there was no significant difference between the groups in percent change in TRACP-5b at any other time point (Table 5, Fig. 2d).

**Table 5:** TRACP-5b changes in the treatment-naïve group and the raloxifene group.

Groups	TRACP-5b (mU/dL)					p
	Percent change in TRACP-5b value from baseline (%)					
	0 M	1 M	3 M	6 M	12 M	
Naïve	446.9 [174, 800]	300.7 [109, 712]⁎1	341.8 [100, 737]⁎2	341.7 [158, 638] ⁎3	303.2 [127, 686] ⁎4	< 0.001
		−29.8 ± 28.7	−20.1 ± 26.3	−16.0 ± 46.2	−20.6 ± 39.7	
RLX	366.9 [169, 752]	233.1 [147, 430]⁎1	273.3 [170, 472]	338.5 [139, 613]	294.8 [108, 441]	0.005
		−32.0 ± 21.3	−20.4 ± 16.3	−3.7 ± 30.1⁎	−15.8 ± 27.7	
P	0.140	0.162	0.291	0.912	0.953	
		0.988	0.715	0.045	0.359	

Data are shown as the mean ± standard deviation and the median [interquartile range].

⁎^1^ : p < 0.05 0 M vs. 1 M.

⁎^2^ : p < 0.05 0 M vs. 3 M.

⁎^3^ : p < 0.05 0 M vs. 6 M.

⁎^4^ : p < 0.05 0 M vs. 12 M; Bonferroni post hoc test after repeated-measures ANOVA.

⁎ P < 0.05; Mann-Whitney U test. *Naïve* treatment-naïve group, *M* month(s), *TRACP-5b* tartrate-resistant acid phosphatase 5b, *RLX* raloxifene group.

### 3.5 Incidence of new fractures

No new fragility fractures occurred during 12 months of treatment with romosozumab.

## 4 Discussion

The results of this study suggest that 12-month treatment with romosozumab may be safe and effective both in female patients with primary osteoporosis who had received prior raloxifene treatment and in those were treatment-naïve. In particular, the increases in lumbar spine BMD at 6 and 12 months were not significantly different between women previously treated with raloxifene and those who were treatment-naïve. Moreover, we did not detect significant differences in changes in BTM levels or the fracture prevention effect of romosozumab between the two groups. The significance of this study is that it is the first to demonstrate the effects of romosozumab following previous raloxifene treatment in women with primary osteoporosis.

The treatment discontinuation rate was 16.1% (*n* = 10) in this study, and one of the common causes was adverse events (*n* = 2, 3.2%). Two non-serious adverse events occurred in the treatment-naïve group. In our previous study, in which 143 treatment-naïve patients were treated with romosozumab, minor adverse events occurred in 2 of 69 patients (2.9%) with primary osteoporosis and in 10 of 74 patients (13.5%) with secondary osteoporosis (Mineta et al., 2024). Romosozumab was relatively safe for women with primary osteoporosis in that study; similarly, in this present study, there were no adverse events during romosozumab treatment following prior raloxifene treatment.

Previous studies have reported an incidence of new vertebral fractures during treatment with romosozumab ranging from 1.2% (Tominaga et al., 2021) to 4.0% (Saag et al., 2017). Although a simple comparison was not possible due to differences in patient background characteristics and the selection criteria used, the incidence of new fractures was 0.0% in the present study and was comparable with that in earlier reports in women with primary osteoporosis. Furthermore, in our previous study (Mineta et al., 2024), which was performed in a real-world setting, we found that the incidence of new fractures was 3.7% in patients who had not been previously treated for osteoporosis, 1.8% in those who had been previously treated with teriparatide, and 3.8% in those who had been previously treated with denosumab. In the same study, BMD increased by 13.0% at the spine and by 4.3% at the total hip after 12 months of treatment with romosozumab in treatment-naïve patients with primary osteoporosis, by 8.7% and 1.9%, respectively, in those who received romosozumab following teriparatide, and by 4.2% and − 0.1% in those who received romosozumab following denosumab. In another study (Messalli and Scaffa, 2010), BMD increased by 18.2% at the spine and by 5.6% at the total hip after 12 months of treatment with romosozumab in treatment-naïve patients with primary osteoporosis, whereas the respective increases were 10.2% and 3.3% in those who received romosozumab following a bisphosphonate. Raloxifene also has antiresorptive activity (Delmas et al., 2002), although its ability to suppress bone resorption is weaker than that of alendronate (Padhi et al., 2014) and denosumab (Sanad et al., 2011). In our present study, BMD increased by 13.5% at the spine and by 4.9% at the total hip after 12 months of treatment with romosozumab in treatment-naïve patients with primary osteoporosis, whereas the respective increases were 16.0% and 3.4% in those who received romosozumab following raloxifene. A novel finding in this study was that previous administration of raloxifene did not appear to attenuate the gain in BMD achieved by romosozumab.

We also examined serum total P1NP as a bone formation marker and TRACP-5b as a bone resorption marker (Nishizawa et al., 2019). Unlike in previous studies, we recorded the median values for both these biomarkers and their percent changes at each time point after starting treatment, given that both influence the effects of treatment with romosozumab following administration of raloxifene. We found the trends in BTMs and their percent changes from baseline on romosozumab for treatment-naïve patients to very similar to those for romosozumab after administration of raloxifene. In patients who received denosumab before romosozumab, P1NP values were low at baseline and the rate of increase from baseline at 1 month was low, but continued to increase gradually thereafter, while TRACP-5b values were low at baseline and the rate of change started to increase from 1 month onwards (Mineta et al., 2024). In patients with a history of treatment with bisphosphonates, although the effect of romosozumab is mild, it causes shifts in BTMs similar to those in patients with a history of treatment with denosumab (Tominaga et al., 2021). Looking at the changes in BTMs in both patients previously treated with raloxifene and treatment-naïve patients, we found that the effectiveness of romosozumab did not appear to be affected by prior raloxifene treatment.

We believe that our new findings would be most relevant in patients requiring a second 12-month course of romosozumab for severe primary osteoporosis (especially those with a lumbar spine BMD < −3.3 SDs). Most patients with osteoporosis may be treated with a single 12-month course of romosozumab followed by sequential treatment with antiresorptive agents. In patients with more severe primary osteoporosis, a second 12-month course of romosozumab may be appropriate. However, sequential treatment with denosumab following a first 12-month course of romosozumab has been reported to attenuate the gain in BMD achieved by a second 12-month course of romosozumab (Kendler et al., 2019). In our present study, sequential treatment with raloxifene following a first 12-month course of romosozumab would not attenuate the effect of a second 12-month course of romosozumab. Furthermore, in another study (Mineta et al., 2025), we demonstrated that the increase in BMD achieved by 12 months of treatment with romosozumab in patients with primary osteoporosis who were previously treatment-naïve was maintained for up to 6 months after switching to raloxifene. Based on the findings of our present study and our previous results, we suggest that sequential treatment with raloxifene for 6 months following a first 12-month course of romosozumab may maintain BMD in patients with severe primary osteoporosis, after which a second 12-month course of romosozumab may provide treatment effects equivalent to those of the first course. We are currently investigating whether a second 12-month course of romosozumab following sequential treatment with raloxifene would be more effective than the previously reported use of a second 12-month course of romosozumab following sequential treatment with placebo or denosumab (Kendler et al., 2019).

This study has some limitations. First, its statistical power may have been weakened by the small sample size. With only 12 patients in the raloxifene group and non-randomized allocation, the study is highly susceptible to confounding and underpowered to detect clinically meaningful between-group differences. Baseline 25-hydroxyvitamin D levels were significantly higher in the raloxifene group, which may have influenced changes in BMD in response to romosozumab. Other unmeasured factors might have differed between the groups as well. The results obtained in this study do not provide definite evidence and merely indicate possibilities. However, the significance of this study is that it is the first to demonstrate the effects of romosozumab following raloxifene. Second, the discontinuation rate was relatively high. Therefore, the safety data may be incomplete. Third, in this study, only symptomatic fractures were evaluated for new fractures, and morphological fractures were not evaluated.

## 5 Conclusions

This study found that 12 months of treatment with romosozumab significantly increased the percent change in spine and total hip BMD at 12 months in primary osteoporosis patients who had received prior treatment with raloxifene and in those who were treatment-naïve. The new fracture rate was very low in patients who received prior raloxifene treatment, suggesting that this approach is safe. Levels of BTMs did not appear to change significantly according to whether there was previous raloxifene treatment. We believe that these findings will lead to novel therapeutic strategies for severe osteoporosis.

## CRediT authorship contribution statement

**Kazuaki Mineta:** Writing – original draft, Visualization, Methodology, Formal analysis, Conceptualization. **Toshihiko Nishisho:** Writing – review & editing. **Masahiko Okada:** Investigation. **Mitsuhiro Kamada:** Data curation. **Koichi Sairyo:** Project administration.

## Funding

None.

## Declaration of competing interest

All authors declare that they have no conflicts of interest.

## Data Availability

The data that has been used is confidential.
